# QM/MM simulations provide insight into the mechanism of bioluminescence triggering in ctenophore photoproteins

**DOI:** 10.1371/journal.pone.0182317

**Published:** 2017-08-04

**Authors:** Maryam Molakarimi, Ammar Mohseni, Majid Taghdir, Zaiddodine Pashandi, Michael A. Gorman, Michael W. Parker, Hossein Naderi-Manesh, Reza H. Sajedi

**Affiliations:** 1 Department of Biochemistry, Faculty of Biological Sciences, Tarbiat Modares University, Tehran, Iran; 2 Department of Biophysics, Faculty of Biological Sciences, Tarbiat Modares University, Tehran, Iran; 3 Australian Cancer Research Foundation Rational Drug Discovery Centre, St. Vincent’s Institute of Medical Research, Fitzroy, Victoria, Australia; 4 Department of Biochemistry and Molecular Biology, Bio21 Molecular Science and Biotechnology Institute, The University of Melbourne, Parkville, Victoria, Australia; Kermanshah University of Medical Sciences, ISLAMIC REPUBLIC OF IRAN

## Abstract

Photoproteins are responsible for light emission in a variety of marine ctenophores and coelenterates. The mechanism of light emission in both families occurs *via* the same reaction. However, the arrangement of amino acid residues surrounding the chromophore, and the catalytic mechanism of light emission is unknown for the ctenophore photoproteins. In this study, we used quantum mechanics/molecular mechanics (QM/MM) and site-directed mutagenesis studies to investigate the details of the catalytic mechanism in berovin, a member of the ctenophore family. In the absence of a crystal structure of the berovin-substrate complex, molecular docking was used to determine the binding mode of the protonated (2-hydroperoxy) and deprotonated (2-peroxy anion) forms of the substrate to berovin. A total of 13 mutants predicted to surround the binding site were targeted by site-directed mutagenesis which revealed their relative importance in substrate binding and catalysis. Molecular dynamics simulations and MM-PBSA (Molecular Mechanics Poisson-Boltzmann/surface area) calculations showed that electrostatic and polar solvation energy are +115.65 and -100.42 kcal/mol in the deprotonated form, respectively. QM/MM calculations and pKa analysis revealed the deprotonated form of substrate is unstable due to the generation of a dioxetane intermediate caused by nucleophilic attack of the substrate peroxy anion at its C_3_ position. This work also revealed that a hydrogen bonding network formed by a D158- R41-Y204 triad could be responsible for shuttling the proton from the 2- hydroperoxy group of the substrate to bulk solvent.

## Introduction

Photoproteins are responsible for light emission in many marine taxa, notably in the coelenterate family. Ca^2+^-regulated photoproteins contain a 2-hydroperoxy coelenterazine chromophore which is tightly but non-covalently bound to the apoprotein. The chromophore is surrounded by four EF-hand loops that provide a hydrophobic cavity for the substrate. Binding of Ca^2+^ to the EF-hands cause conformational changes resulting in oxidative decarboxylation of coelenterazine and returning the excited coelenteramide to its ground state [1]. This mechanism causes light to be emitted in the blue range of the spectrum [2, 3].

**Fig 1:**
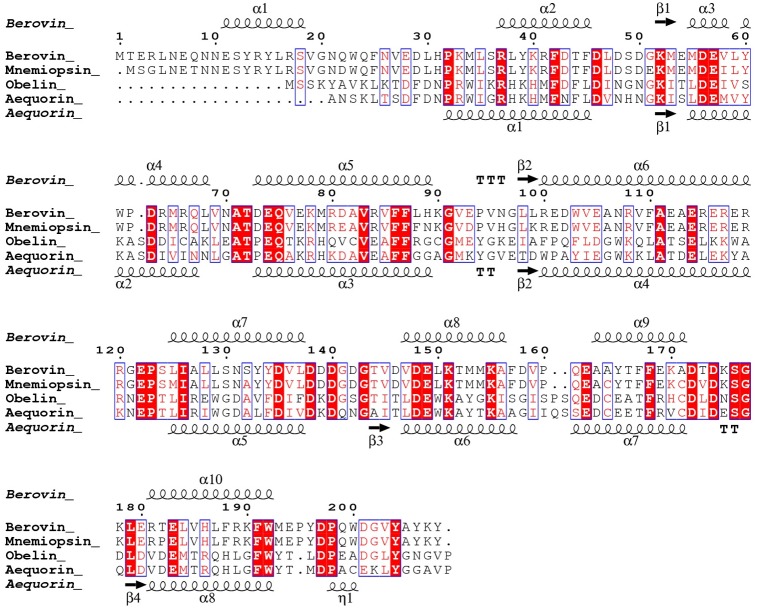
Multiple sequence alignment of mnemiopsin (GQ231544) and berovin (pdb ID: 4MN0) with aequorin (pdb ID: 1EJ3) and obelin (pdb ID: 1EL4). The secondary structure elements of berovin (top) and aequorin (bottom) are indicated. The figure was made using ESPript3 [25]. A comparison between aequorin and berovin showed 29% of sequence identity.

Crystal structures of a number of coelenterate photoproteins have been determined including aequorin, obelin and clytin; in apo [4], coelenterazine bound [5–8] and coelenteramide bound [9–12] states. These studies revealed a hydrophobic substrate-binding pocket that not only contains hydrophobic side-chains, but also hydrophilic aromatic residues (e.g. H16, Y132, H169 and Y184 (aequorin numbering) [2, 3, 13–17]). Although members of the ctenophore photoprotein family, such as berovin, bolinopsin, mnemiopsin and B fosPP, have been well studied [18–21], the only published crystal structure is that of apo-berovin, bound to calcium and magnesium [22, 23]. Comparison of the structures of apoberovin with apoobelin and apoaequorin in the Ca^2+^-loaded form, show that despite a low degree of sequence identity between ctenophore and coelenterate photoproteins (Fig 1), they have a high degree of structural similarity [24]. Although the bioluminescence reaction in both photoprotein families appears similar [24], the arrangement of amino acid residues around coelenterazine in the ctenophore family, the mechanisms of reaction initiation and light emission remain largely unknown.

The location of the substrate-binding site in the crystal structure of berovin has been predicted based on the presence of a similarly sized cavity to that observed in the crystal structures of obelin and aequorin. The predicted coelenterazine-binding site of berovin is mainly hydrophobic, like the coelenterate family, but the amino acid residues surrounding the binding site are very different. There are many His, Trp and Tyr residues in berovin sequence, however, only two amino acid residues Y204 and W192 are found in the same position to key residues in the coelenterate family [26]. The equivalent residue of Y204 in aequorin (Y184) is responsible for initiating the light emission reaction. However, the Y204F mutation in berovin retains complete activity whereas the Y184F mutation in aequorin reduced its activity to 14% [27].

A recent study on berovin has proposed a catalytic mechanism suggesting that the substrate is bound to the internal cavity of ctenophore photoproteins as a 2-peroxy anion, so that its negatively charged peroxy anion is stabilized through a coulomb interaction with the positively charged guanidinium group of R41 which is paired with Y204. The binding of Ca^2+^ to berovin was suggested to cause some conformational changes leading to the bioluminescence reaction and destroying the charge-charge interaction network that stabilizes the peroxy anion in the cavity. However, it's assumed that the formation of peroxy anion is involved in proton transfer in coelenterate photoproteins and the conformational changes will be sufficient for triggering the reaction [28].

Since uncertainty still largely exists about the catalytic mechanism in the ctenophore family, we investigated the binding mode of coelenterazine, in protonated (2-hydroperoxy) and deprotonated (2-peroxy anion) states, to berovin through induced fit docking (IFD) and molecular dynamics (MD) simulations. Estimated pKa values of Y204 were obtained from the last snapshots of both MD simulations, using the CpHMD method. Then, the snapshots taken at the stable period from the MD trajectory were collected for energetic analysis using MMPBSA and normal mode calculations. We then calculated the QM/MM reaction energy profile from the results of the MD simulations. These results guided site-directed mutagenesis studies to identify key amino acid residues involved in the reaction.

## Materials and methods

### Initial structure preparation

The crystal structure of berovin (pdb ID: 4mn0) [26] was used as input for the computational studies. Using the I-Tasser server [29–31], missing residues 1–2, 27–35, 194–208 were added. I-TASSER threads the protein sequence through the PDB structure library and searches for the possible alignments, uses ab initio modeling for unaligned regions. Then, the resultant model was refined by molecular dynamics (MD) simulation using the AMBER12 software package [32] with ff03 force field [33].

### Docking studies and analysis of the complexes

The berovin complexes of coelenterazine, in the protonated and deprotonated states, were constructed by IFD as implemented in the Schrödinger Suite [34]. The crystal structure of the apo form had too narrow a cavity to accommodate coelenterazine indicating that the protein must undergo substantial conformational changes during ligand binding. IFD accounts for both ligand and protein flexibility by iteratively combining flexible ligand docking into a rigid protein and flexible protein structure prediction. IFD first uses softened-potential force fields (with scaling factor of 0.5) to dock a flexible ligand into a rigid protein binding pocket with the Glide program [35]. To proceed, the best predicted binding pose is utilized for protein structure prediction *via* the refinement module of the Prime program, in which any residues having at least one atom within 5 Å of the ligand are treated as flexible, while all other residues are held fixed. Each refined protein structure is ranked by total Prime energy, and the top-ranked protein structure is then used for re-docking of the ligand with the Glide program [35], using a hard (conventional) potential function without scaling.

### Molecular dynamics simulations

The final best-ranked complexes from IFD were minimized and used for molecular dynamics simulations. The AMBER12 package [32] was used for preparation of the berovin-coelenterazine complexes based on the docking studies. The Antechamber module was applied to calculate the AM1-BCC charges of the two states of coelenterazine. Then, ff99SB [36, 37] and GAFF force fields [38] were used to model the complex with the addition of sodium ions for neutralizing the system. The resulting complexes were solvated with TIP3P water molecules in a truncated octahedron periodic box with an 8 Å radius buffer zone of water molecules around the complexes. The systems were neutralized by adding the corresponding number of counterions (Na^+^) using the LEaP module. Then, the systems were energy minimized for 50000 cycles, using the steepest descent algorithm together with the conjugate gradient method to remove any bad contacts between atoms. The minimized systems were first gradually heated to 300 K over 100 ps using a Langevin thermostat [39] in a constant condition (NVT). Then, system is equilibrated for 100 ps at constant pressure (1 atm) with a 2 fs time step to adjust the periodic box size in a constant condition (NPT). Finally, 70 ns unrestrained production simulations were conducted for each system using an integration time step of 2.0 fs. During the production stage, every 500 time steps coordinates were saved and used for energy and structure calculation. All the MD simulations were done employing periodic boundary conditions with a 10 Å cutoff for non-bonded interactions, and long-range electrostatics interactions were carried out adhering to the particle mesh Ewald (PME) method [40]. The SHAKE algorithm [41] was used to fix all covalent bonds involving hydrogen atoms.

### Binding free energy calculations

Multiple snapshots were generated from the production phase of MD simulations. For every snapshot, after stripping off the water molecules and counter ions, the free energy was calculated for the protonated and deprotonated complexes. The binding free energy was computed as:

$$\Delta G_{bind}=\;\Delta E_{gas}+\Delta G_{solv}-\;T\Delta S$$

Where the interaction energy of protein-ligand complex in gas phase (Δ*E*_*gas*_) is given by

$$\Delta E_{gas}=\Delta E_{elec}+\Delta E_{vdw}$$

While the protein-ligand electrostatic and van der Waals interactions are represented by Δ*E*_*elec*_ and Δ*E*_*vdw*_, respectively.

The solvation free energy is divided into a polar and a nonpolar part, where Δ*G*_*solv-pol*_ is the electrostatic contribution to the solvation energy and Δ*G*_*solv-nonpol*_ is the nonpolar solvation term:

$$\Delta G_{solv}=\Delta G_{solvpol+}\Delta G_{solvnonpol}$$

In this study, the polar contribution was calculated by solving the Poisson-Boltzmann (PB) equation [40] as implemented in AMBER12 [32].

The non-polar solvation energy Δ*G*_*solv-nonpol*_ was calculated from the solvent-accessible surface area (SASA) using the hard-sphere atomic model. The probe radius of the solvent was set to 1.4 Å. The atomic radii for the solute were taken from the PARSE parameter set [42]. Δ*G*_*solv-nonpol*_ is determined using:

$$\Delta G_{solvnonpol}=\gamma \left(SASA\right)\;+\beta$$

Where the surface tension γ and the offset β were set to the standard values of 0.00542 kcal mol^−1^ Å^−2^ and 0.92 kcal/mol, respectively [42].

TΔS, the change of solute entropy upon complexation, was computed from normal mode analysis using the NMODE module of the AMBER12 program. To obtain a detailed view of the substrate binding, the interaction energies were further decomposed into contributions from each residue of berovin in the two complexes.

### QM/MM simulation

The QM/MM simulations were performed using the AMBER12 program [32]. Final complex configurations resulting from the classical MD simulations were used as initial structures. The QM/MM simulations were carried out using the PM6 method [43, 44] implemented in AMBER12. QM parts consisted of ligand molecule and side-chains of R41 and Y204 around the peroxy group of the substrate in deprotonated and protonated states. Hydrogen link atoms [45] were placed between Cγ and Cδ on R41 and between Cβ and Cγ on Y204. The QM region consists of 80 atoms for both QM/MM simulations. The rest of the protein, water molecules and (Na^+^, Cl^-^) ions were considered as the MM region. The MM part was defined by AMBER ff99SB force fields [36, 37]. This system was subjected to a QM/MM simulation for 300 ps and 250 ps in protonated and deprotonated systems, respectively.

### General setup and parameters of the MD simulation for the pKa calculation

For the constant pH molecular dynamics (CpHMD), it needs to provide relative files and describe the types of residues for titrating. The available titratable residues in AMBER are aspartate, glutamate, histidine, lysine, tyrosine and cysteine, which are all defined as described by Mongan et al [46]. At the first, titrating residues were renamed in order to prepare the pdb files [46, 47]. Using the LEaP module of AMBER12, hydrogen atoms were added [32]. Subsequently, the topology and coordinate files of the systems were generated with LEaP. The CpHMD and all other MD simulations were performed using Sander of AMBER12 and the ff99SB force field [36]. *cpin* file was generated using the *cpinutil*.*py* program of AMBER12 molecular dynamics package. In the *cpin* file all possible titrating residues were defined. In all simulations the implicit solvation model of generalized Born model (igb = 2 in AMBER), was employed and salt concentration (Debye-Huckel based [48]) and non-bonded interactions cutoff were set on 0.1 and 30 Å, respectively. The systems were minimized for 50000 cycles, using the steepest descent algorithm together with the conjugate gradient method. During minimization, all of the hydrogen atoms were minimized while all heavy atoms were restrained with a constant force of 10 kcal/(mol∙Å^2^) to their initial positions.

For the NVT ensemble, 400 ps of MD were conducted to heated system from 10 to 300 K while the non-hydrogen atoms of the protein were restrained with a constant force of 2 kcal/(mol∙Å^2^). Then, for the NPT ensemble, 500 ps MD simulation with no position restraints was carried out to equilibrate the structure and stabilize the density. In this step, the temperature was maintained at 300 K and other parameters remained unchanged. In the implicit solvent method, the constant pH was switched on and the solvent pH was set to a starting pH of 7.0. Moreover, the bond involved hydrogen atoms were constrained with the SHAKE algorithm and the time step was set on 2 fs. Finally, the CpHMD method [46, 47] was used to simulate berovin from a pH 7.0 environment to pH 14 environment. During simulation, Y204, D158 and K207 was allowed to change its protonation state. Protonation state change attempts were made every 200 fs. The remaining parameters were identical to those described in the previous section.

### Reagents and bacteria

*cp*-coelenterazine and Ni-NTA agarose were purchased from Resem BV (The Netherlands) and Qiagen (Qiagen, Hilden, Germany), respectively. *Dpn*I was purchased from Fermentas (Fermentas, Vilnius, Lithuania) and *E*. *coli* BL21 (DE3) was obtained from Novagen (Madison, WI, USA). Kanamycin and Isopropyl-D-thiogalactopyranoside (IPTG) were purchased from Invitrogen (Carlsbad, CA, USA). PCR purification kit and plasmid extraction kit were provided from Bioneer (Bioneer, South Korea). All the other necessary chemicals such as 8-anilino-naphthalene-1-sulfonic acid (ANS), Tris and CaCl_2_ were purchased from Merck (Darmstadt, Germany). All measurements were repeated at least three times.

### Construction of mutants

To create mnemiopsin mutants, Quick Change Site-Directed Mutagenesis was performed by using pET28a expression vector containing apomnemiopsin 1 gene (GenBank accession No. GQ231544) [20]. Amplification of DNA was done by sequential steps: denaturation at 95°C for 30 sec; 12 cycles of 95°C for 30 sec; 55°C for 1 min; 68°C for 5 min. The obtained fragments were digested with *Dpn*I and subsequently were transformed into *E*. *coli* BL21 (DE3). The plasmids of mutations were verified by DNA sequencing.

### Protein expression and purification

Transformed *E*. *coli* BL21 (DE3) strains were cultivated in a medium of Luria-Bertani broth and kanamycin (50 μg ml ^−1^) at 37°C and subjected to a reciprocal shaking (250 rpm min^−1^) for 12–14 h. To inoculate the production medium, 1% (v/v) concentration of pre-cultured cells were added to 250 ml of terrific broth (TB) medium in 1 L Erlenmeyer flasks, and after achieving an OD600 of 0.6–0.8, protein expression was induced with 1 mM IPTG. The cultivation was continued for 6 h at 27°C with reciprocal shaking (250 rpm min^−1^) then, using a centrifuge at 5000 ×g for 20 min, the cells were harvested and the pellet.

In order to purify of wild type (WT) and mutant proteins, cell pellets were resuspended in 50 mM of Tris, pH 8.0 containing 40 mM imidazole, 1 mM PMSF, 300 mM NaCl and 10% (v/v) glycerol and were subsequently disrupted with ultrasound on ice, then centrifuged at 12000 ×g for 20 min at 4°C. Ni-NTA resin was used for the purification of His6-tagged photoprotein. The supernatant was applied to a column equilibrated in 50 mM Tris buffer, pH 9.0, 300 mM NaCl and 40 mM imidazole. The column was washed by the same buffer and the adsorbed proteins were eluted by elution buffer (50 mM Tris buffer, pH 9.0, 300 mM NaCl and 250 mM imidazole). The purified proteins were subjected to protein concentration measurement by the Bradford method, SDS-PAGE analysis and bioluminescence activity measurements. Collected fractions were dialyzed against 50 mM Tris buffer, pH 9.0 containing 1 mM EDTA, 150 mM NaCl, 8 mM ammonium sulfate and 10% (v/v) glycerol by gentle stirring for 6 h at 4°C.

### Preparation of semi-synthetic photoproteins and determination of luminescence activity

The purified proteins were diluted in 50 mM Tris buffer, pH 9.0 containing 10 mM EDTA and were mixed with a given volume of *cp*-coelenterazine at a final concentration of 8 μM. The mixtures were briefly vortexed and placed at 4°C in the dark for 16 h. In order to determine the luminescence activities of the semi-synthetic photoproteins, 10 μl of the regenerated mixtures were added to 40 μl of 50 mM Tris-base buffer, pH 9.0 and the solution was placed in a luminometer (Sirius tube luminometer, Berthold Detection System, Germany). Then 50 μl of 50 mM Tris buffer containing 40 mM CaCl_2_, pH 9.0 was added to the sample solution and luminescence intensity was measured.

## Results and discussion

### Three dimensional structure prediction

The 3D structure of apo-berovin (pdb ID: 4MN0) [26] was used to investigate which amino acid residues are involved in the bioluminescence mechanism of the ctenophore family. Due to lack of some residues in apo-berovin structure, it is of vital importance to obtain an accurate structure of the apo-berovin prior to investigate of bioluminescence mechanism. Missing residues 1, 2, 27–35 and 194–208 were added based on mitrocomin (4nqg) and berovin (5bpj) as templates using the I-Tasser server. Model 1 was selected as best predicted model with C-score 0.11, TM-score 0.73 ± 0.11, and RMSD 5.2 ± 3.3 Å. The Ramachandran plots of best selected model were acquired from PROCHECK. The PROCHECK Ramachandran plot showed 80.5% residues in most favored regions and 17.1% residues in additional allowed regions i.e., the total of 97.6% residues in allowed regions which showed the reliability of model. The resultant model was refined by molecular dynamics (MD) simulation.

### Prediction of coelenterazine binding mode in berovin

**Fig 2:**
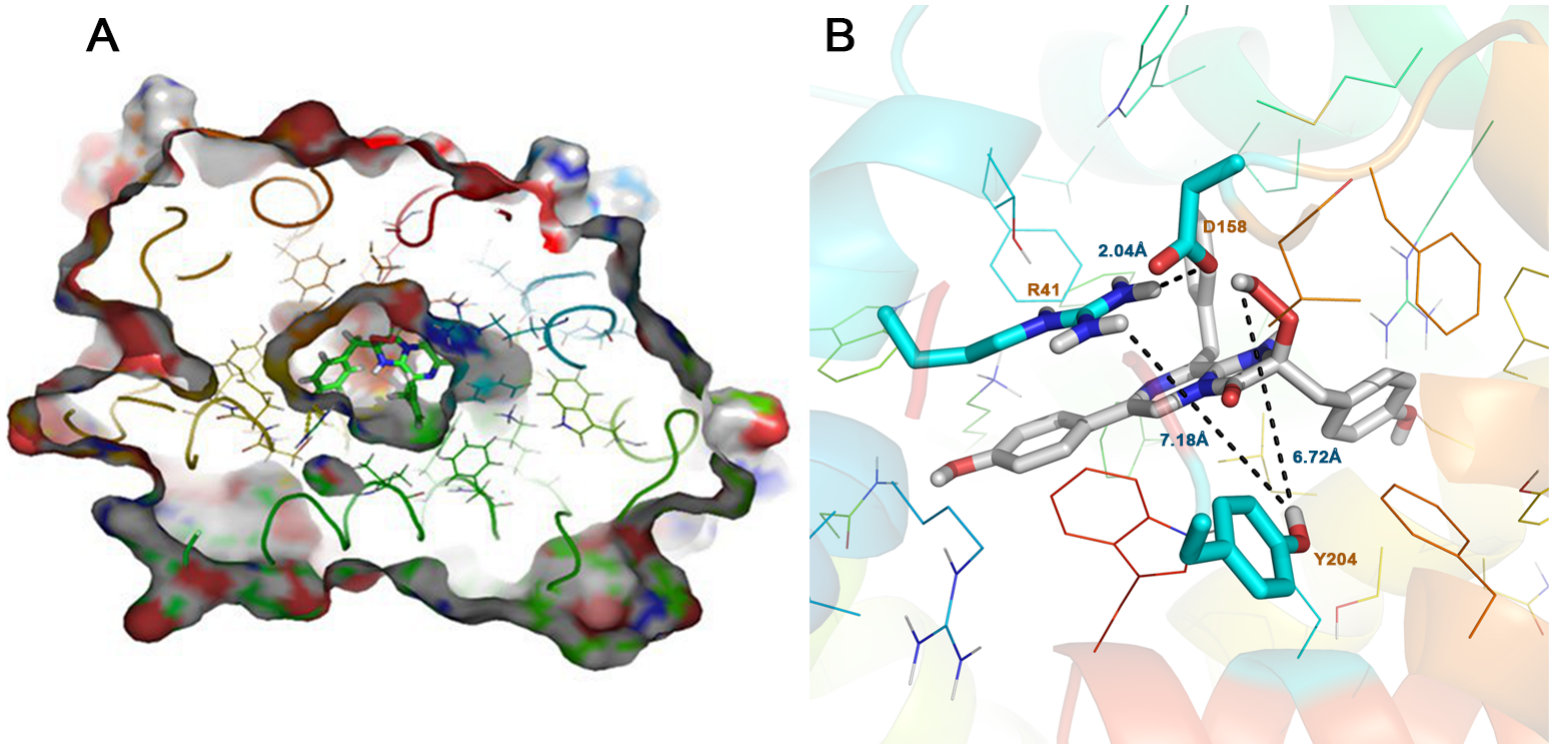
Induced fit docking study on substrate binding mode. A) Cutaway view of the berovin cavity showing coelenterazine occupied in. B) Close-up of the amino acid residues around the 2- hydroperoxy group of coelenterazine.

It is challenging to predict the binding pose of coelenterazine based on the crystal structure as the putative binding pocket was too small to fit the large substrate. To obtain the binding pose, the induced fit docking (IFD) protocol, as implemented in the Schrödinger Suite [34], provided a plausible docking solution. Burakova *et al*., based on the crystal structure and the impact of mutations on berovin bioluminescence, suggested that coelenterazine is bound as a 2-peroxy anion adduct [28]. Thus, the binding mode of coelenterazine was determined in both protonated and deprotonated states (Fig 2). The two states adopted a similar binding mode and were stabilized by stacking and van der Waals contacts with non-polar residues, mostly phenylalanine, and by interactions with polar side-chains.

### Dynamic stability of the complexes

**Fig 3:**
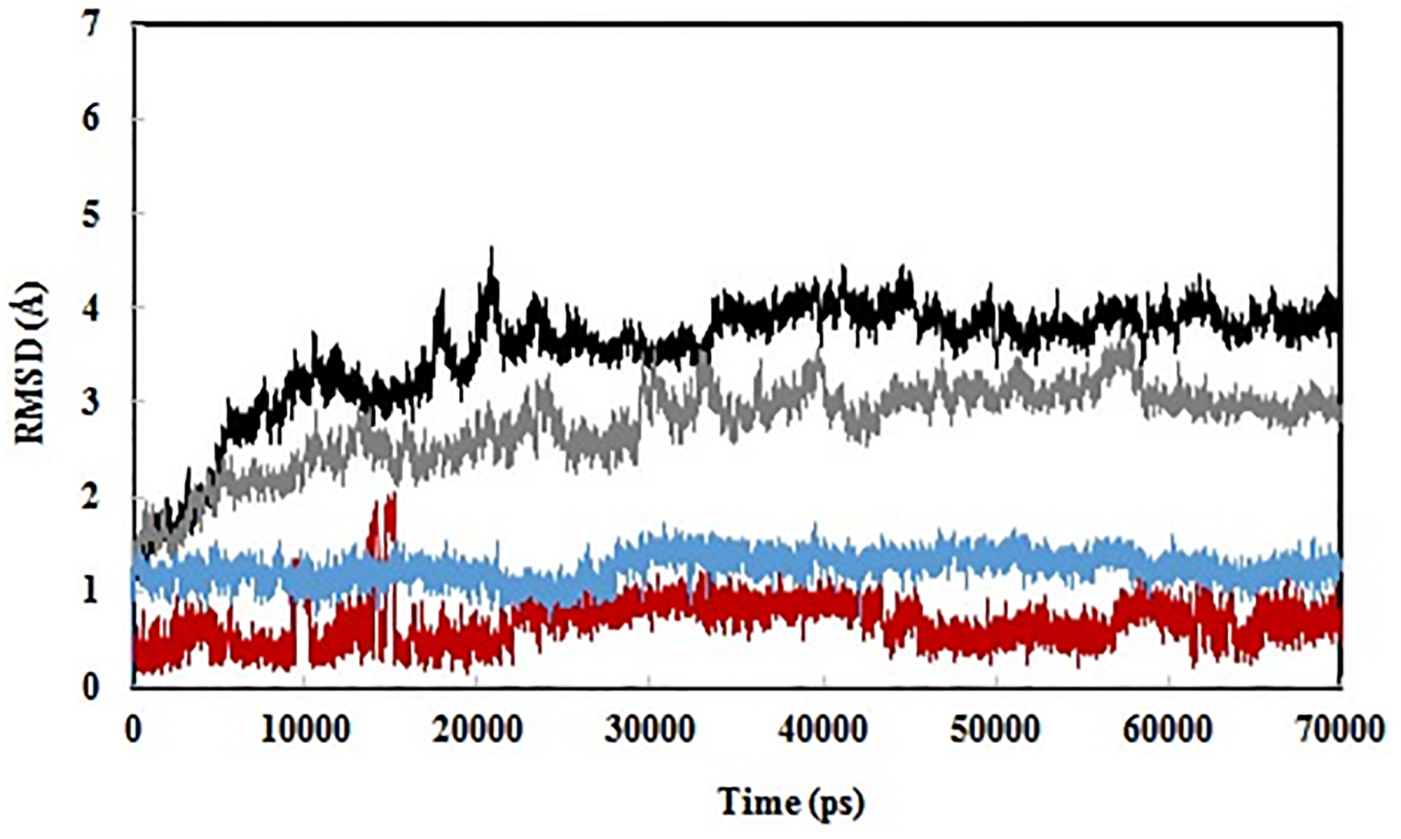
MD simulations of the berovin complexes. Root-mean-square deviation (RMSD) of the backbone Cα atoms of the berovin in the protonated (black) and deprotonated (gray) states with respect to the first snapshot during the simulations as a function of time. RMSD of the coelenterazine are also shown in the protonated (red) and deprotonated (blue) states.

Two 70 ns MD simulations were performed to investigate the dynamic stability of the protonated and deprotonated substrate-bound states. Root-mean-square deviation (RMSD) values for backbone Cα atoms of the proteins were monitored relative to the starting structures over the entire simulations (Fig 3). RMSD curves showed that the backbone trajectories of structures were stable, reaching equilibrium after the first 30 ns of the simulation. For two complexes, from 30 ns until the end of the simulations, the values fluctuated very little, indicating equilibrated protein structures. RMSD values of coelenterazine over the entire simulations were also shown in Fig 3. As shown, there were no significant structural changes of coelenterazine in two complexes, indicating appropriate orientation of coelenterazine in its binding site. The final models were used for subsequent structural analyses.

**Fig 4:**
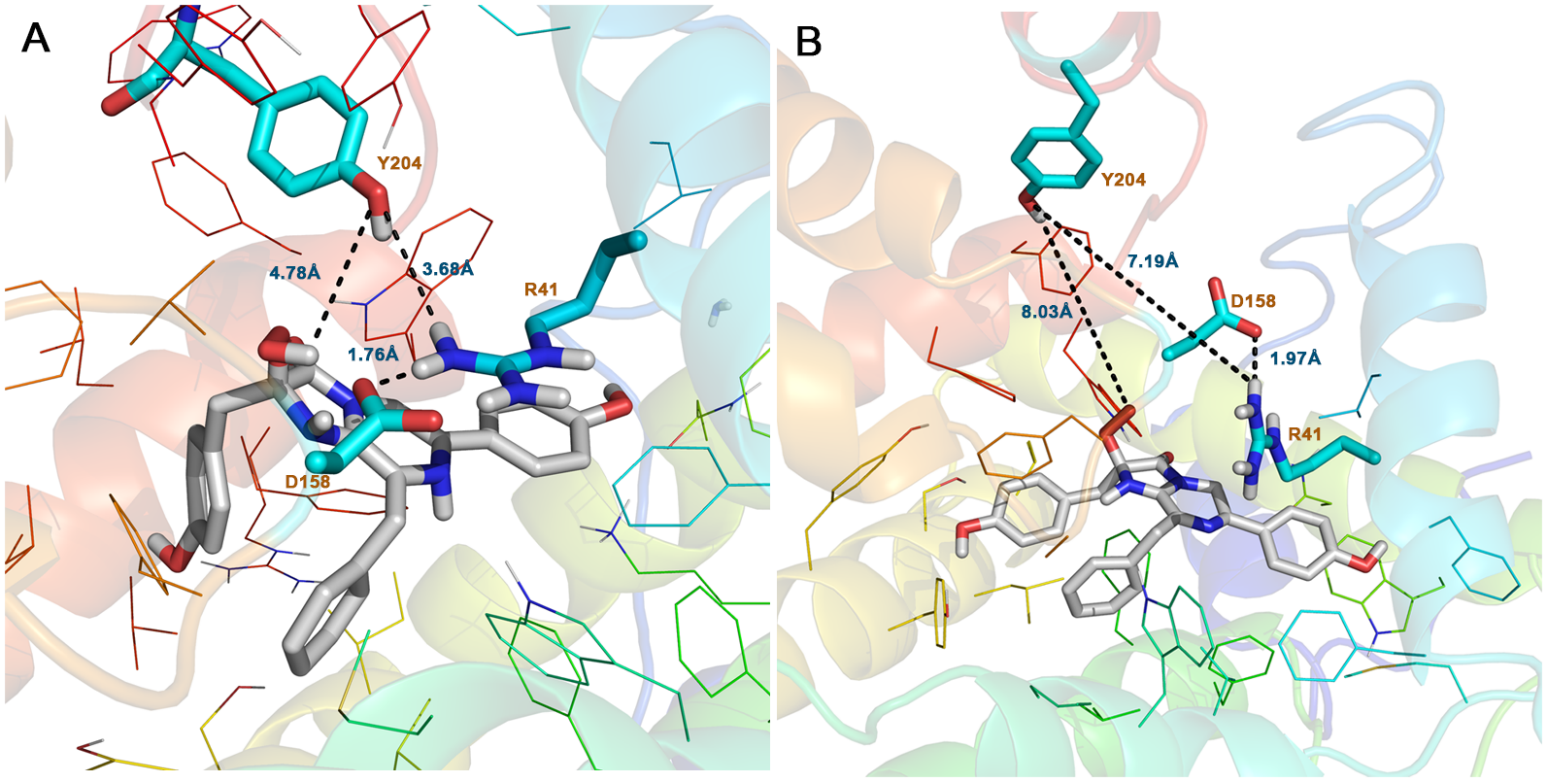
Key interactions of the colenterazine substrate and residues surrounding the substrate peroxy group. The structure is taken from the last snapshot of the molecular dynamics simulations. A) Protonated form of substrate and B) deprotonated form of substrate. Hydrogen bonds are labeled in black dashed lines.

A detailed analysis of the MD trajectories corresponding to the protonated state of the complex suggested that the following residues are pivotal for the stability of the substrate in the cavity: R41, F45, W61, M65, M79, F86, F87, K90, W103, N107, L129, S130, Y133, F157, V159, D158, F167, L184, L187, F188, W192, Y204 and Y206. Many of these residues provide an appropriate reaction environment through hydrophobic interactions, thus stabilizing coelenterazine. Indeed, the coelenterazine-binding site of berovin is very hydrophobic, as found in the coelenterate family but does contain some hydrophilic residues. R41, D158, W192 and Y204 were observed around the 2- peroxy group of substrate and to participate in hydrogen bonding interactions with it (Fig 4). Furthermore, the 2-*ρ*-hydroxybenzyl group of coelenterazine is stabilized through hydrogen bonding with S130 amino acid residue. In addition, N107, K90 and W103 amino acid residues are located surround the 6-*ρ*-hydroxybenzyl group.

Mechanistic and structural studies of the coelenterate photoproteins indicate that their substrate-binding pocket also contain hydrophilic residues such as H16, Y132, H169 and Y184 (aequorin numbering). Three His-Tyr-Trp triad have been found to be critical for catalysis and the stability of the chromophore in the binding site and it seems that the histidine residues are responsible for the transmission of the proton between substrate and protein [2, 27, 49, 50]. Comparison of the structures of Ca^2+^-loaded apoberovin with Ca^2+^-loaded apoaequorin shows a low degree of sequence identity between the ctenophore and coelenterate families and thus efforts to identify an equivalent catalytic triad in substrate-binding sites of berovin and mnemiopsin have been unsuccessful.

A number of mutagenesis studies have been carried out on berovin and mnemiopsin in order to determine the key amino acid residues that surround the coelenterazine [28, 51–53]. However, these studies are incomplete based on the computational results described above so further mutations were performed here in order to obtain a complete picture. In all, 13 mutations were performed on mnemiopsin, mostly targeting polar and aromatic residues, and their specific activity measured in comparison with the WT variant (Table 1). The loss of luminescence activity in these mutants show that each of these residues is critical for the reaction and their arrangement provide a stable environment for substrate binding in the ctenophore family.

**Table 1:** Relative specific activities of some mutations on berovin and mnemiopsin in comparison with their WT.

Type of mutant (previous study)	Relative activity (%)	Reference
Berovin (WT)	100	
Y204F	100	Ref. [26]
Y134H	56.2	Ref. [28]
W61F	3.7	Ref. [28]
K90M, R41M, K90E, Y133F, Y133H, M154Y, M154Q, W192F, Y204E, Y204K, Y134F	<1	Ref. [26, 28]
Mnemiopsin (WT)	100	
R41K	900	Ref. [53]
S130G	71.6	Ref. [52]
V185T	46.6	Ref. [52]
W103Y	15	Ref. [51]
W103F	6	Ref. [51]
M153Y	5	Ref. [51]
M79H, R41M, R41E	<1	Ref. [51,53]
K207R	75.5	present study
Y206F	58.2	present study
Y208F	41.7	present study
D158N	25	present study
K207M	8.7	present study
Y204F	5.9	present study
L38H, R41H, W61K, N107W, Y133F, L129W, F188H	<1	present study

Some mutations were performed in this study, the specific activity of WT mnemiopsin was 40.0 × 10^6^ RLU/s mg protein.

### Binding free energy analysis of substrates

Since the RMSD values of structures were stable after the 30 ns production run, the snapshots collected in the stable period were used for the energetic analysis. The total binding energies and the individual energy components are summarized in Table 2. The ∆G_bind_ for the protonated and deprotonated forms was -10.83 and -24.23 kcal/mol, respectively. A detailed analysis of individual energy components showed that the van der Waals term is the most important driving force for substrate-binding for each state. In the protonated state, the polar solvation energy opposes the binding term, whereas the nonpolar solvation term, which corresponds to the burial of solvent-accessible surface area (SASA) upon binding and the electrostatic energy, contributes slightly favorably to the binding energy. In the deprotonated state the electrostatic and polar solvation energy are +115.65 and -100.42 kcal/mol, respectively. This is due to the replacement of the charge-charge interactions of the protein-ligand complex during MD simulation with water. Therefore, the loss of electrostatic stabilization in the deprotonated state results in a positive electrostatic binding contribution, the charged binding site and ligand are more easily solvated and so the polar solvation contribution is observed as a negative value.

**Table 2:** Results of free energy calculation of berovin complexed with coelenterazine in two states by MM-PBSA and NMODE.

	∆G_vdw_	∆G_elec_	∆G_solv-polar_	∆G_solv-nonpol_	∆G_MMPBSA_	T∆S	∆G_calc_
Protonated form	-55.41	-49.62	73.62	-5.15	-36.57	-25.73	-10.83
Deprotonated form	-58.79	115.65	-100.42	-4.96	-48.53	-24.30	-24.23

All energies are in kcal/mol.

To obtain a detailed picture of ligand-residue interactions, a set of residues at the binding site were selected for decomposition analysis (Table 3). Coelenterazine in the protonated state has a binding free energy ~ 6 kcal/mol more favorable than the deprotonated form. The favorable residues can be classified into hydrophilic and hydrophobic categories. The hydrophobic residues make strong van der Waals interactions with substrate. Most of these hydrophobic residues, which are centered in the binding pocket (including L38, F45, W61, F86, W103, N107, L129, F157, F188 and W192), made the most favorable contributions to the substrate binding. The loss of catalytic activity when L38, W61, N107, L129, F188 and W192 (Table 1) are mutated can be attributed to the reduced binding potency caused by the loss of hydrophobic contacts.

**Table 3:** Energy contributions from individual residues to substrate binding. All energies are in kcal/mol.

Residues	Protonated form	Deprotonated form
L-38	-0.13	0.00
R-41	-1.82	-9.91
F-45	-0.21	-1.32
W-61	-0.84	-1.57
F-86	-0.58	-1.19
F-87	-0.24	-1.22
K-90	-0.18	-0.24
W-103	-0.29	-0.58
N-107	-0.04	-0.02
L-129	-1.73	-2.42
S-130	-0.62	-1.16
Y-133	-0.22	-1.12
F-157	-2.12	-2.06
D-158	0.23	-5.76
F-188	-3.23	-0.34
W-192	-3.26	-1.28
Y-204	-0.46	0.01
Y-206	-0.005	0.00
K-207	0.08	-0.01
Y-208	-0.45	0.01
Ligand	-21.56	-15.87

The hydrophilic residues can form strong hydrogen bonding and electrostatic interactions with the substrate. The charged residues (R41, R66, K90, D158 and K207) made favorable contributions to binding free energy mainly through electrostatic interactions. Polar residues including S130, Y133, Y204 and Y208 made the favorable contributions to binding free energy, mainly through hydrogen interactions. As shown in Table 1, the decrease of the electrostatic interactions could be result in the decrease of activity in the D158N variant in mnemiopsin. Mutation of Y204, equivalent to Y184 in aequorin, to phenylalanine did not affect the bioluminescence activity of berovin but decreased the bioluminescence activity significantly in mnemiopsin. The K205M and K205R mutants reduced the activity by 8.7% and 75.5%, respectively which suggests that the positive charge in this position has an important role in luminescence emission.

### The impact of alkaline pH on protonated state of peroxide group

**Fig 5:**
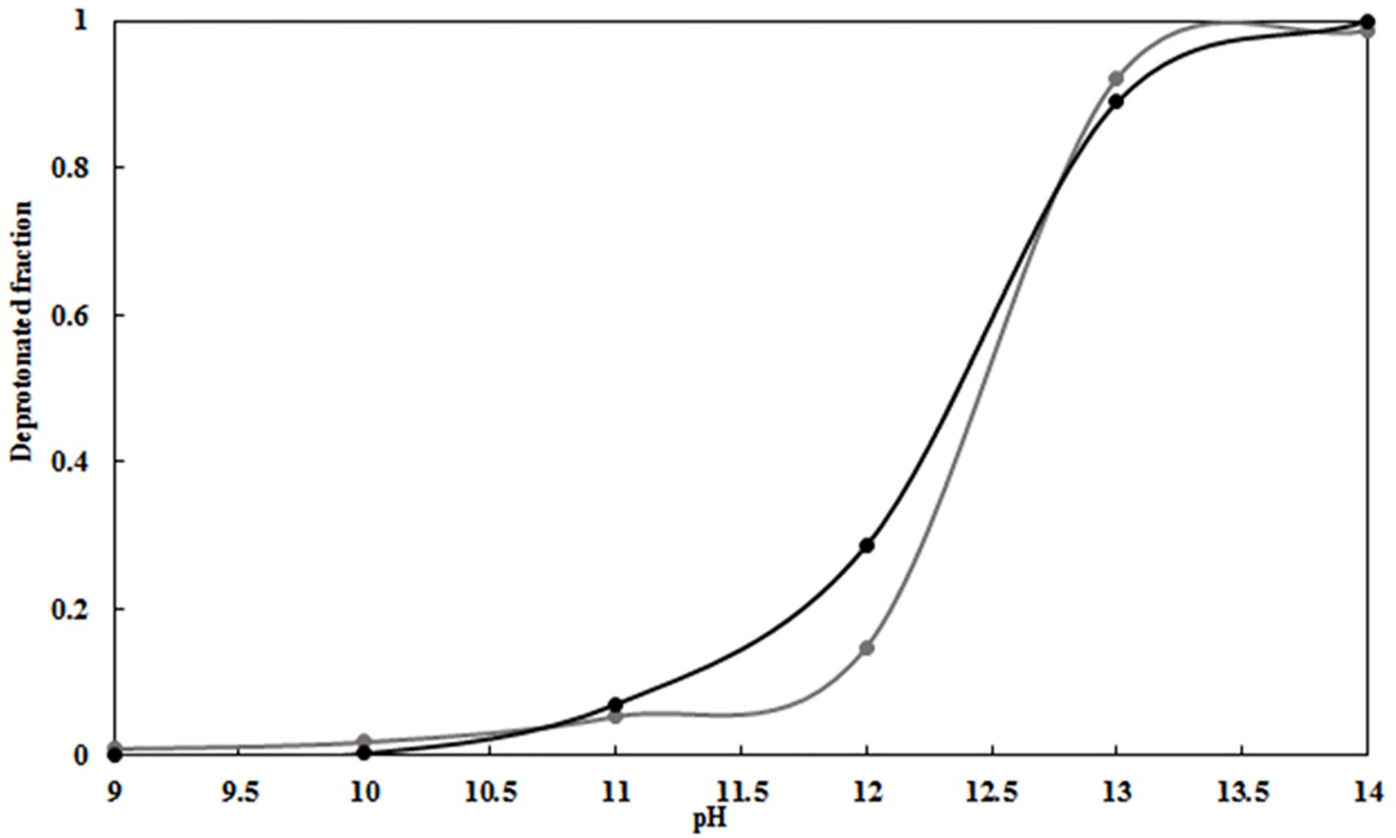
Titration curves forresidue Y204 in berovin. CpHMD simulation starting at pH 9.0 for the protonated (black) and deprotonated (gray) state of the coelenterazine.

Apo-berovin is efficiently converted into an active photoprotein at pH 9.0, in contrast to cnidarian photoproteins. Burakova *et al* suggested that although the intrinsic pKa of the hydroxyl group of tyrosine is 10.2, the pKa value of the Y204 within berovin may be much lower due to the presence of the nearby positively charged guanidinium group of R41 [28]. In the present study, we have devised the CpHMD method to investigate the effect of alkaline pH on ionization of the side-chain groups of amino acids. The pKa values of Y204 were calculated for the protonated and deprotonated states of coelenterazine and are summarized in Fig 5. The results show that the pKa value for Y204 in both states is above 10.

On the other hand, proton shuttling between tyrosine and hydroperoxide group for oxidative decarboxylation process occur when the hydroperoxide group in coelenterazine and the tyrosine have similar pKa values, thus it is reasonable to suggest that pKa values of hydroperoxide is above 10 [2]. Since the ctenophore family exhibit bioluminescence activity at pH 9.0, it can be concluded that the tyrosine residue and the hydroperoxide exists in a protonated state in the cavity. Our structural analysis suggests that the hydroxyl group of Y204 is located next to the 2- peroxy group of coelenterazine and thus the reduction of catalytic activity to ~5% in the Y204F mutant of mnemiopsin is due to the critical role of Y204 in the catalytic process. In addition, the hydroxyl group ionization state of Y204 is also very important for oxidative decarboxylation process.

### QM/MM studies on the reaction mechanism

**Fig 6:**
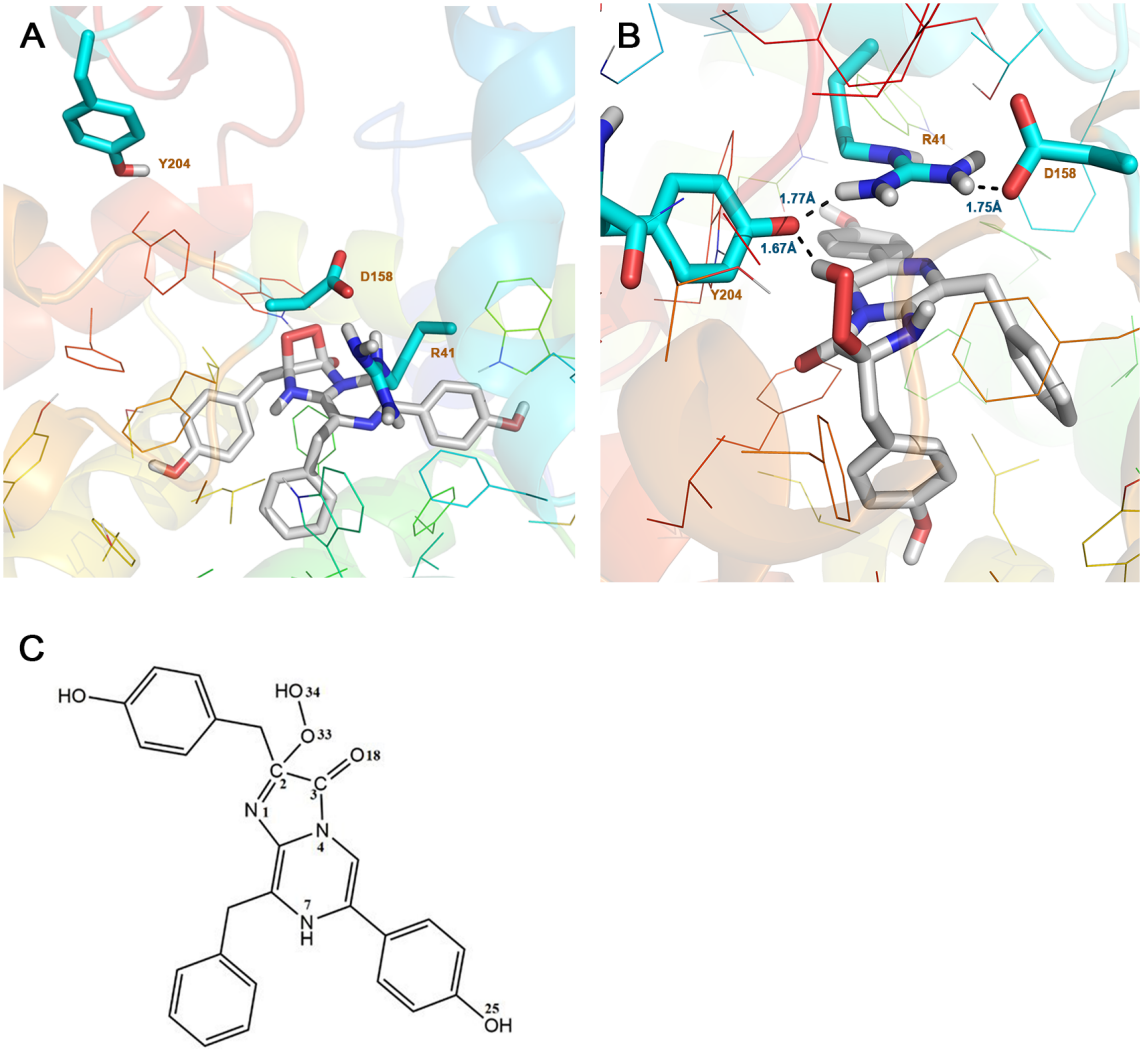
Close-up views of the coelenterazine binding site from the last snapshot of the QM/MM. A) Deprotonated form of coelenterazine: dioxetane intermediate formed with nucleophile attack of peroxy anion of coelenterazine to its C_3_. B) Protonated form of coelenterazine: hydrogen bond network around of proxy group formed with catalytic triad. Distances are shown in black lines. C) Molecular structure of coelenterazine.

Based on the model of Burakova *et al*, the substrate’s negative charge within the substrate-binding cavity of ctenophore photoproteins is bound as a 2-peroxy anion adduct of coelenterazine, which is stabilized by the positive charge of a guanidinium group of R41 paired with Y204. The bioluminescence reaction of ctenophore photoproteins is triggered by conformational changes due to binding of calcium ions that leads to the destruction of the charge—charge interaction network that stabilizes the peroxy anion. To obtain further insights into the mechanism, QM/MM calculations were performed on the deprotonated complex. Considering the importance of R41 for the stability of the coelenterazine’s hydroperoxy group, side chains of R41, Y204 and coelenterazine were selected in the QM layer. In comparison with the initial structure, the most important change was the nucleophilic attack of the peroxy anion of coelenterazine onto its C_3_ resulting in a dioxetane intermediate (Fig 6), so that the distance of the C_3_-O_34_ bond is decreased from 2.80 to 1.34 Å. Moreover, the C_2_-C_3_ bond of coelenterazine is elongated from 1.50 Å to 1.60 Å and the distance of the N_4_-C_3_ bond is increased from 1.31 to 1.49 Å (Table 4). This could be result in the break of N_4_-C_3_ bond and decarboxylation of coelenterazine [54]. For the reliability of these results, three other snapshots were extracted from the MD trajectory of deprotonated complex and selected for QM/MM calculation. However, the formation of dioxetane intermediate was observed for all of these snapshots. The QM/MM analysis, together with the pKa calculation above, provides strong evidence in support of destabilization of the deprotonated form of the substrate in the cavity.

**Table 4:** Selected bond lengths (Å) of coelenterazine obtained from the MD simulation and from the QM/MM studies of the deprotonated form.

	MD simulation	QM/MM simulation
O_34_-C_3_	2.80	1.34
N_4_-C_3_	1.41	1.58
C_2_-C_3_	1.50	1.60

**Fig 7:**
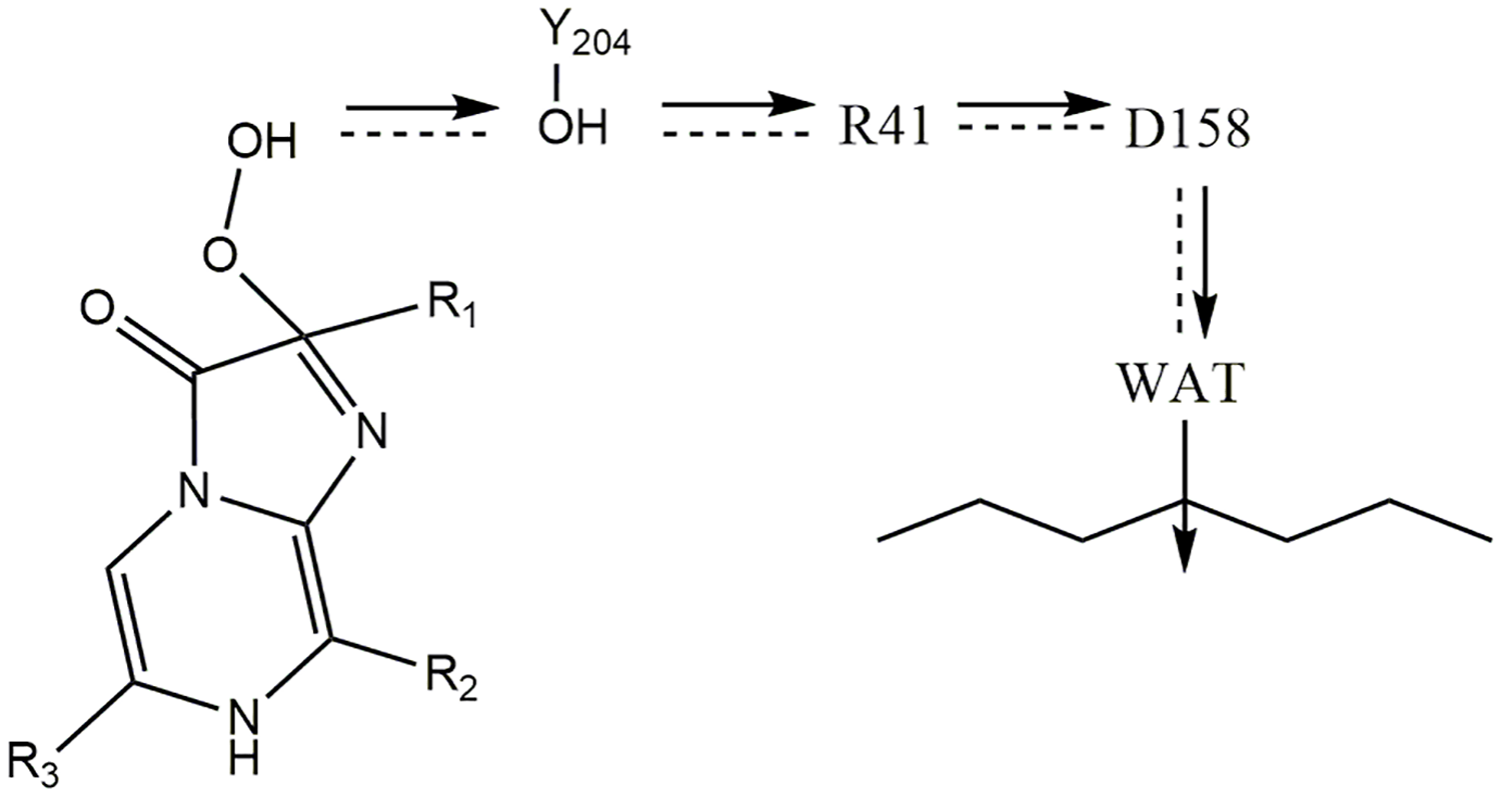
Suggested mechanism for initiating of the reaction in the ctenophore family. A D158-R41-Y204 triad around the 2- hydroperoxy group of coelenterazine forms a hydrogen-bonded network that could shuttle a proton from the 2- hydroperoxy group to bulk solvent.

In a second QM/MM calculation, the protonated form of coelenterazine and side chains of R41, Y204 were considered as a QM region, with the rest of the complex a MM region. Upon minimization with the AMBER ff99SB force field, the side-chain of Y204 moved close enough to be in hydrogen bonding distance of the 2- hydroperoxy group of the substrate. Studies by McCapra and Chang [2] on the coelenterate family show that some intermediates are engaged in the oxidative decarboxylation of coelenterazine [2]. Reformation of the anion peroxy occurs in the presence of Ca^2+^ and with the help of the Tyr-Trp-His catalytic triad. In this mechanism, the binding of Ca^2+^ causes a conformational change, which makes the hydrogen bond of H175-Y190 shorter and stronger. As a result, H175 and Y190 become protonated and nucleophilic, respectively, with the tyrosine being protonated by the hydroperoxy group of coelenterazine and produces a peroxy anion [2]. For this reason, in the QM/MM calculations, this tyrosine residue was manually deprotonated to form a tyrosinate anion. In an optimized structure, the Y204 form strong hydrogen bond (1.67 Å) with an oxygen atom of the 2- hydroperoxy group, which indicates that proton is ready to be transferred to Y204 (Fig 6). The distance between R41 and Y204 amino acids side chain is 1.77 Å. Notably, the side-chain of D158 is only 1.75 Å away from the side-chain of R41. The activity measurements in Table 1 suggest that R41, D158 and Y204 are all engaged in the reaction mechanism. Indeed, a D158-R41-Y204 triad could form a hydrogen-bonded network that could shuttle a proton from the 2- hydroperoxy group to bulk solvent [55]. In support of this suggestion, an R41M replacement leads to loss of activity compared to WT berovin (Table 1). A reasonable explanation for the loss activity of R41M mutant might be that the methionine residue is not capable of forming a hydrogen bond required for its role in facilitating proton transfer in both directions. Thus, R41 may be regarded as a general base. Additional support for this suggestion is found in mutation studies of the Y204 position (Table 1). For example, the replacement Tyr204 to Phe leads to a drop in activity compared to WT and suppression of a direct proton transfer pathway between the 2- hydroperoxy group of coelenterazine and Y204F can account for this effect. In addition, the substitution of Asp158 to Asn preserves 25% of bioluminescence activity compared to WT (Table 1). These results, as depicted in Fig 7, suggest that a proton can transfer from the 2- hydroperoxy group of coelenterazine to solvent with the assistance of Y204 and R41.

## Conclusions

QM/MM calculations of the berovin-coelenterazine complex supplemented by MM-PBSA analysis, and theoretical pKa calculations have led to testable predictions of the key amino acid residues that stabilize the coelenterazine as well as a catalytic triad that are essential for initiating catalytic activity in the ctenophore family. The predictions are supported by extensive mutagenesis studies reported here and elsewhere (Table 1). A recent study on berovin has proposed a mechanism suggesting that the substrate is bound to the internal cavity of ctenophore photoproteins as a 2-peroxy anion and stabilized through a charge-charge interaction with R41. The binding of Ca^2+^ was proposed to destroy this charge-charge interaction and trigger the light emission mechanism. To test this model, we determined the coelenterazine orientation in the berovin binding site and key stabilizing residues for the two states of the peroxy group in coelenterazine: protonated and deprotonated. To obtain the binding pose, the IFD protocol was implemented for both of these states. To obtain an estimate of the dynamic stability of the protonated and deprotonated complexes, 70 ns MD simulations were performed. MM-PBSA was then applied in order to get an estimate of the binding energy of berovin-coelenterazine in both states. Results from MM-PBSA analysis show that electrostatic and polar solvation energy are +115.65 and -100.42 kcal/mol in the deprotonated form, respectively and the van der Waals term is the most important driving force for substrate binding in each complex. Interestingly, the electrostatic and polar solvation energy of the deprotonation state make negative contributions to the substrate binding. In addition, pKa calculations for Y204 showed that the pKa value in both states are above 10. Since the ctenophore family show bioluminescence activity at pH 9.0, it is reasonable to assume that the peroxy group exists in a protonated state in the cavity, since the hydroperoxide group in coelenterazine and the tyrosine have similar pKa values. QM/MM calculations were performed for two complexes. In deprotonated form, the most important change is that the nucleophilic attack of peroxy anion of coelenterazine to its C_3_ produces a dioxetane intermediate. QM/MM, MMPBSA and pKa analysis provide evidence that the deprotonated form of substrate in binding site is probably unstable. The QM/MM results on the protonated state suggest that R41, D158 and Y204 are all engaged in the reaction mechanism. A D158-R41-Y204 triad creates a hydrogen-bonded network that could shuttle a proton from the 2- hydroperoxy group to bulk solvent. In fact, site-directed mutagenesis studies, R41M, D158N and Y204F, confirm the pivotal role of this triad in the reaction mechanism (Table 1). A summary of the proton shuttling mechanism is shown in Fig 7. In this study, we only discussed about D158-R41-Y204 triad that is equivalent to H169-Y184-W173 triad and could play important role for triggering bioluminescence. Finding the other triads such as H16-Y82-W86 in ctenophore family needs more study. Confirmation of this mechanism awaits the determination of a 3D atomic structure of a ctenophore-substrate complex.

## Supporting information

S1 Text PDB file for modeling structure of apo-berovin. (PDB)

S2 Text PDB file for apo-berovin after 10 ns MD simulation. (PDB)

S3 Text PDB file for berovin-coelenterazine complex obtained from molecular docking (protonated state). (PDB)

S4 Text PDB file for berovin-coelenterazine complex after 70 ns MD simulations (protonated state). (PDB)

S5 Text PDB file for berovin-coelenterazine complex after 70 ns MD simulations (deprotonated state). (PDB)

S6 Text PDB file for berovin-coelenterazine complex obtained from QM/MM calculation (protonated state). (PDB)

S7 Text PDB file for berovin-coelenterazine complex obtained from QM/MM calculation (deprotonated state). (PDB)

S8 Text PDB file for modeling structure of apo-mnemiopsin. (PDB)
